# Increasing engagement with an occupational digital stress management program through the use of an online facilitated discussion group: Results of a pilot randomised controlled trial

**DOI:** 10.1016/j.invent.2017.08.001

**Published:** 2017-08-24

**Authors:** Stephany Carolan, Peter R. Harris, Kathryn Greenwood, Kate Cavanagh

**Affiliations:** a School of Psychology, University of Sussex, Falmer BN1 9QH, United Kingdom; b Research and Development Department, Sussex Partnership NHS Foundation Trust, United Kingdom

**Keywords:** CAU, Care as usual, CBT, Cognitive behavioural therapy, DG, Discussion group, MSG, Minimal support group, RCT, Randomised controlled trial, WLC, Wait list control, ITT, Intention to treat, CI, Confidence intervals, CBT, Internet, Online, Web-based, Randomised controlled trial, Stress, Work

## Abstract

**Introduction:**

Rates of work-related stress, depression and anxiety are high, resulting in reduced work performance and absenteeism. There is evidence that digital mental health interventions delivered in the workplace are an effective way of treating these conditions, but intervention engagement and adherence remain a challenge. Providing guidance can lead to greater engagement and adherence; an online facilitated discussion group may be one way of providing that guidance in a time efficient way. This study compares engagement with a minimally guided digital mental health program (WorkGuru) delivered in the workplace with a discussion group (DG) and without a discussion group (MSG), and with a wait list control (WLC); it was conducted as a pilot phase of a definitive trial.

**Methods:**

Eighty four individuals with elevated levels of stress from six organisations were recruited to the study and randomised to one of two active conditions (DG or MSG) or a WLC. The program WorkGuru is a CBT based, eight-week stress management intervention that is delivered with minimal guidance from a coach. Data was collected at baseline, post–intervention and at 16-week follow-up via online questionnaires. The primary outcome measure was number of logins. Secondary measures included further engagement measures, and measures of depression, anxiety, stress, comfort and enthusiasm. Quality measures including satisfaction and system usability were also collected.

**Results:**

A greater number of logins was observed for the DG compared with the MSG; this was a medium between group effect size (*d* = 0.51; 95% CI: − 0.04, 1.05). Small to medium effect size differences were found at T2 in favour of the active conditions compared with the control on the DASS subscales depression, anxiety and stress, and the IWP subscales enthusiasm and comfort. This was largely maintained at T3. Satisfaction with the intervention was high with individuals in the MSG reporting greater satisfaction than individuals in the DG.

**Conclusions:**

This study shows that access to an online facilitated discussion group increases engagement with a minimally supported occupational digital mental health intervention (as defined by the number of logins), but that this doesn't necessarily result in improved psychological outcomes or increased satisfaction when compared to access to the intervention without the group. Access to the web-based program was associated with lower levels of depression, anxiety and stress and an increase in comfort and enthusiasm post intervention; these changes were largely maintained at follow-up.

**Trial registration:**

This trial was registered with ClinicalTrials.gov on March the 18th 2016 NCT02729987 (website link https://clinicaltrials.gov/ct2/show/NCT02729987?term=NCT02729987&rank=1).

## 1 Introduction

In the UK prevalence rates for work-related stress, depression and anxiety are high, accounting for 11.7 million lost working days (HSE, 2016) and resulting at both a clinical (Birnbaum et al., 2010, Dewa et al., 2007, Dewa and Hoch, 2015, Sanderson and Andrews, 2006) and a sub clinical level (Martin et al., 1996) in reduced work performance and absenteeism. There is evidence that these conditions are both preventable and treatable in the workplace. A recent meta-analysis has shown that digital mental health interventions delivered in the workplace can be effective at reducing psychological distress and increasing workplace effectiveness (Carolan et al., 2017); however, despite examples of occupational digital mental health interventions that have achieved good adherence (Ebert et al., 2016, Heber et al., 2016, Thiart et al., 2015, Umanodan et al., 2014) one of the challenges of digital mental health still remains increasing adherence and engagement (Cavanagh and Millings, 2013, Eysenbach, 2005, Kohl et al., 2013). While digital interventions are typically designed for widespread accessibility, uptake can be low and the discontinuation curve steep. A randomised controlled trial (RCT) of a digital mental health intervention delivered in the workplace reported that only 5% of participants started one or more of the modules (Boiler et al., 2014), and a trial of digital mindfulness delivered in a workplace reported that between 42% and 52% of all participants in the active conditions never logged on to the program (Allexandre et al., 2016). Carolan et al. (2017) found that the mean highest reported completion across 19 studies in their meta-analysis was 45% with a range of 3% to 95%.

Research has consistently shown that providing guidance can lead to greater adherence to web-based interventions (Andersson and Cuijpers, 2009, Brouwer et al., 2011, Baumeister et al., 2014, Hilvert-Bruce et al., 2012, Mohr et al., 2011). An online facilitated discussion group may be one way of providing that guidance in a time efficient way. Previous studies (Andersson et al., 2005, Berger et al., 2011, El Alaoui et al., 2015) have incorporated discussion groups into their interventions but have failed to identify the impact of the group on the effectiveness of the intervention.

In this study we therefore compare engagement with a minimally supported CBT based digital mental health program (WorkGuru) delivered in the workplace with and without access to a facilitated discussion group, and to a wait list control (WLC), and explore whether increased engagement suggests increased effectiveness. The trial was conducted as a pilot trial to gain greater confidence in predicting effect size, refining optimum engagement of the intervention (adherence), understanding accuracy of engagement measures, and understanding the challenges of conducting the trial in the workplace.

## 2 Methods

### 2.1 Trial design

A three-arm randomised controlled trial was conducted comparing a minimally supported web-based CBT based stress management intervention (WorkGuru) delivered with and without an online facilitated bulletin board, with a wait list control (WLC). Randomisation was conducted on a ratio of 1:1:1. All participants had unrestricted access to care as usual (CAU). The trial was conducted to examine the effect of an online facilitated discussion group on engagement with a minimally supported digital stress management intervention delivered to employees, and to look at the estimated potential effectiveness of the program. Assessment took place at baseline (T1), at post treatments (8 weeks, T2) and at follow-up (16 weeks after randomisation, T3). Participants in the active conditions completed a credibility and expectancy questionnaire at two weeks following randomisation. All assessments were completed online.

This trial was conducted and reported in line with the CONSORT eHealth checklist (Eysenbach and CONSORT EHEALTH group, 2011). Further information about this trial is available from the trial protocol (Carolan et al., 2016). The study was approved by the University of Sussex Science and Technology Cross-School Research Ethics Committee (reference number ER/SC587/1), and registered with ClinicalTrials.gov NCT02729987.

### 2.2 Participants and procedure

UK based organisations that had subscribed to the WorkGuru mailing list were invited to participate in this study. Participating organisations circulated a statement to staff inviting them to follow a link or contact the first named author (SC) for more information. Participating organisations were encouraged to offer employees a minimum of 1 h a week over the eight-week period to complete the program. Participants who were: i) aged 18 or over, ii) employed by a participating organisation, iii) willing to engage with a web-based CBT based stress management intervention, iv) had access to the Internet, v) had access to a tablet or computer, vi) had an elevated level of stress, as demonstrated by a score of ≥ 20 on the PSS-10 (Cohen et al., 1983), were recruited to the study between March and June 2016. No exclusion criteria were set. The cut off of 20 on the PSS-10 represents one standard deviation (6.53) above the mean (13.02) in a large (*n* = 2387) US general population sample (Cohen and Williamson, 1988). Participants who met the inclusion criteria were invited to complete a baseline questionnaire that was completed online. A consent statement was included on the front page of the questionnaire; participants gave consent to take part in the study by completing the questionnaire. Participants were informed that their participation was confidential and their organisation would not be informed of which employees were participating in the study. On completion of the baseline questionnaire, participants were randomised to one of the three study arms. An allocation schedule was created using a computer generated randomisation sequence (random.org). An independent researcher allocated each group (A, B, or C) as an active condition (with or without a facilitated bulletin board) or the WLC. The study researchers were blind to the group allocation. Participants allocated to the Minimal Support Group (MSG) were able to access the intervention immediately. Participants allocated to the discussion group were also able to access the intervention immediately, but were asked to wait for up to three weeks for the start of the group. The delay in starting the facilitated group was to enable an optimum number of participants to begin the group together; participants were encouraged to access the bulletin board and take part in an introductory exercise while they were waiting for the group to start. Participants allocated to the WLC were able to access the intervention after 16 weeks.

### 2.3 Intervention

A more detailed description of the web-based CBT based stress management program WorkGuru is available from Carolan et al. (2016). The program was presented on a secure platform that participants logged-on to using an email address and a self-generated password. The eight-week program was based on the psychological principles of CBT, positive psychology, mindfulness and problem solving. It consisted of seven core modules that all participants were encouraged to complete and three additional modules. The core modules included information and exercises on stress, resilience, values, cognitive restructuring, automatic thoughts, unhelpful thinking styles and time management. The additional modules contained information on mindfulness, problem solving and imagining the future self. Participants completed the modules at their own pace. They could either complete a questionnaire and receive suggestions of which modules that they might find useful, or choose the modules that they wished to complete themselves. The modules consisted of a combination of educational reading, audio, short animations and interactive exercises. Participants could also complete eight self-monitoring standardised questionnaires, including the Perceived Stress Scale (Cohen et al., 1983), the Subjective Happiness Scale (Lyubomirsky and Lepper, 1999), and the Brief Resilience Scale (Smith et al., 2008). They were also able to opt-in to a weekly motivational email (the “Monday Morning Message”) that contained a motivational quotation and advice on staying well in the workplace, and could set themselves email reminders to visit the site. To encourage engagement, an e-coach contacted the participants through the site when they first logged-on, at two weeks, and at six weeks. Messages from the coach were all personalised. Participants could choose to share work with the coach and could contact the coach for information or advice. The coach responded within 24 h.

While using the WorkGuru site, users were prompted to contact their GP, NHS 111 or the Samaritans if they were concerned about their mental health. Contact details for NHS 111 and the Samaritans were given.

#### 2.3.1 Minimal support group (MSG)

Participants allocated to the MSG had access to the intervention as described above.

#### 2.3.2 Online discussion group (DG)

Participants allocated to the discussion group had access to the intervention as described above; they also had access to an eight-week online guided discussion group that was delivered via a bulletin board. Each week the coach introduced one or more of the modules and encouraged discussion about the topic. Participants chose a user name, and were able to be anonymous in the group.

### 2.4 Measurements

#### 2.4.1 Primary outcome measure

The primary outcome measure was engagement, which was measured using the number of logins to the site. The number of logins was chosen as the primary outcome measure because it is the most commonly reported objective exposure measure used in studies of digital health (Brouwer et al., 2011, Donkin et al., 2011).

#### 2.4.2 Secondary outcome measures

Secondary measures included further measures of engagement (the number of modules completed, the number of page views, self-reported engagement measures using one-item on a 5-point Likert scale with a range of 0 to 5), and of psychological outcomes: a measure of depression, anxiety and stress (DASS-21) and a measure of wellbeing at work (IWP). DASS-21 (Lovibond and Lovibond, 1995) is a 21-item scale that was designed to measure the negative emotional states of depression, anxiety and stress. Items are answered on a 4-point Likert scale (0 = did not apply to me at all; 3 = applied to me very much or most of the time). Cronbach's α for the subscales at baseline were: depression α = 0.88; anxiety α = 0.90; stress α = 0.84 in this study. The IWP Multi-Affect Indicator (Warr, 1990) is a measure of wellbeing at work. It is a 16-item scale that is scored on a 7-point scale. Participants are asked the approximate amount of time they have felt different emotions during the week (0% of the time = never; 100% of the time = always). The subscales for depression and anxiety are reverse scored, resulting in higher scores representing higher wellbeing. Cronbach's α for the subscales at baseline were: enthusiasm α = 0.87; anxiety α = 0.90; comfort α = 0.74; depression α = 0.84 in this study.

#### 2.4.3 Other measures

Other measures taken were: client satisfaction (CSQ; Larsen et al., 1979), which is an eight-item questionnaire that is rated on a 4-point scale with reverse scoring on four items. The questionnaire was developed to assess general satisfaction with services, α = 0.95 in this study; acceptability (adapted from Schneider et al., 2012) which is a six-item questionnaire that is rated on a five-point scale (1 = strongly disagree; 5 = strongly agree), α = 0.62 in this study; treatment credibility and patient expectancy (CEQ; Devilly and Borkovec, 2000), which is a six-item questionnaire that utilises two rating scales, one from 1 to 9 and the other from 0 to 100%. Participants are asked what they thought or felt about the treatment. The measure achieved α = 0.92 in this study; system usability (Brooke, 1996), which is a ten-item questionnaire, rated on a five-point scale (1 = strongly disagree; 5 = strongly agree). Five of the items are reverse scored, and the sums of the scores are multiplied by 2.5 to obtain an overall value. A score of < 50 would be regarded as a cause for significant concern; scores above 70 are seen as acceptable, with scores in-between suggesting the need for continued improvement (Bangor et al., 2008). In this study α = 0.92; negative effects of treatment, using one-item developed for this study, which asks the question: “What, if any, positive or negative effects caused by the program/being in the control group did you experience?” Possible moderators explored were: goal conflicts, using the goal conflict index developed for this study. This is a three-item questionnaire that is rated on a five-point scale (1 = strongly disagree; 5 = strongly agree), α = 0.59; job autonomy, using the nine-item autonomy subscale from the Work Design Questionnaire, (Morgeson and Humphrey, 2006), which is rated on a five-point scale (1 = strongly disagree; 5 = strongly agree), Cronbach's alpha for the subscales at baseline were all α ≥ 0.83 in this study; time perception (Etkin et al., 2015) a 5-item questionnaire, which is rated on a five-point scale (1 = strongly disagree; 5 = strongly agree), α = 0.74 in this study; levels of psychological distress at baseline as measured on DASS.

Engagement measures specific to the discussion group were taken (number of views of the bulletin board and the number of contributions) as well as the Online Support Group Questionnaire (Chang et al., 2001), which is a nine-item questionnaire that is rated on a ten-point scale (1 = not at all; 10 = very much). Cronbach's alphas for the subscales were α > 0.77 in this study. Existing psychological illness, CAU, sickness absence for stress related complaints, and contamination between the groups were monitored. Demographic measures included age, gender, fluency of written and spoken English, country of birth (UK, non-UK), relationship status, work role, number of working hours (low, middle, high), organisation, education level, income bracket and familiarity with the online environment.

### 2.5 Statistical analyses

All analyses were performed using SPSS version 22 (IBM, 2013). Due to the pilot nature of this study descriptive information was presented; exploratory inferential analyses were conducted using ANCOVA and *t*-test as appropriate. Analyses of the primary and secondary outcome measures were conducted on an intention-to-treat basis; sensitivity analysis included a per-protocol analysis. Per-protocol was defined as three or more logins to the WorkGuru site. A significance level of 0.05 (two-sided) was used for all analyses. Cohen's *d* using pooled standard deviations, and 95% CIs were calculated. Effect sizes were interpreted using the classification given by Cohen (small = 0.2, medium = 0.5, large = 0.8; Cohen, 1988). Outliers > 3.29 standard deviations away from the mean were identified (Field, 2013). Missing data was imputed using the Last Observation Carried Forward method. Baseline differences between groups were explored using chi-square and ANOVA (as appropriate).

## 3 Results

### 3.1 Recruitment and participants

Individuals (*n* = 780) who had subscribed to a WorkGuru marketing mailing list while attending conferences were invited to nominate their organisation to take part in the research. Nineteen organisations expressed an initial interest; none of which had previous experience of WorkGuru. Six of the organisations were recruited into the study. All six organisations were UK based: two were local authorities, two were universities, one was a third sector organisation, and one was a telecommunication organisation. Participating organisations directed staff to information and promoted the study through emails, intranet, in-house magazines and newsletters. The marketing statement used by the organisations gave a brief description of the intervention and emphasised that participation would be entirely confidential.

**Fig. 1:**
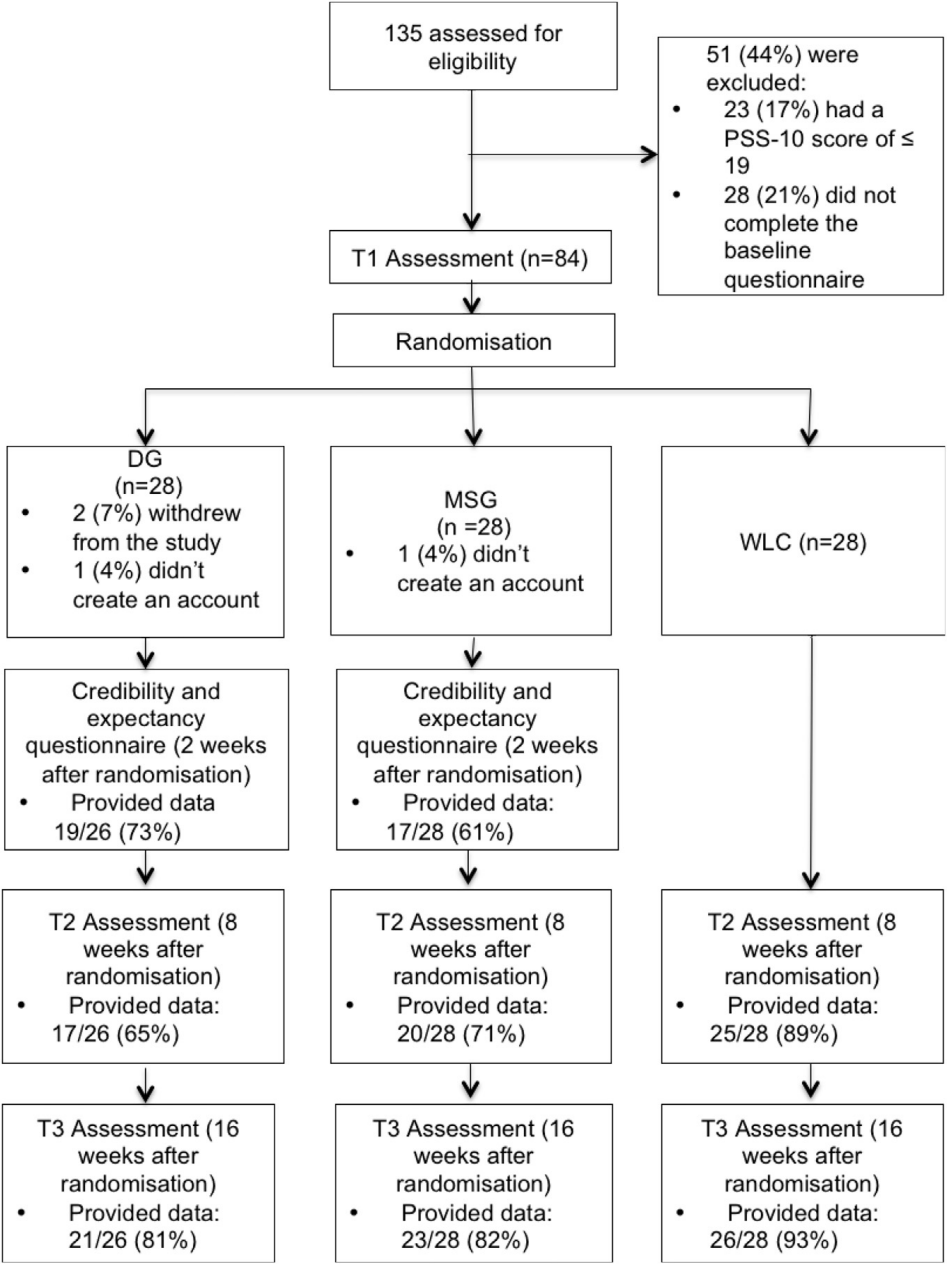
Flow of participants.

Fig. 1 summarises the recruitment and flow of participants through the study. Of the 135 individuals who were assessed for eligibility, 23 were excluded because they scored ≤ 19 on PSS-10, and 28 were excluded because they did not compete the baseline measure. A total of 84 individuals were randomised. Two individuals (2.4%) withdrew from the study after randomisation: one reported changing jobs and the other reported an increase in workload, which meant he/she would not have time to participate in the study.

For all the engagement measures (logins, number of pages visited, modules completed), the data was gathered through the web-based program. Two participants did not create an account for themselves, resulting in data being available for 80 of the 82 participants (97.6%). Of the 82 participants, 62 (75.6%) completed questionnaires at 8 weeks after randomisation (T2), and 70 (85.4%) 16 weeks after randomisation (T3). Of the 54 participants in active conditions, 36 (66.7%) completed the credibility and expectancy questionnaire 2 weeks after randomisation. Chi-square tests found the groups did not differ in regard to missing data (all *p* > 0.10). Participants who provided data at T2 and T3 did not differ from those who did not on baseline scores of depression, anxiety of stress, or on gender or allocated group.

### 3.2 Baseline characteristics

Demographic data for all study participants are displayed in Table 1. A significant difference was found between the randomised groups on both the occupation (*p* = 0.013) and the highest qualification (*p* = 0.009) variables. Sensitivity analysis was run with highest qualification as a covariate; no effect was found. No other differences were found between the groups on demographic information or levels of depression, stress or anxiety at baseline. Mean levels of depression, anxiety and stress for participants at baseline, as measured on the DASS, were moderate to severe for depression (M = 20.2, SD = 9.6) and moderate for both anxiety (M = 12.3, SD = 8.1) and stress (M = 23.8, SD = 8.3; Lovibond and Lovibond, 1995).

**Table 1:** Demographic information.

	Total *n* = 82	DG *n* = 26	MSG *n* = 28	WLC *n* = 28
Demographic characteristics				
Gender, female (%)	70 (85)	21 (81)	24 (86)	25 (89)
Mean age (SD)	41.0 (10.2)	40.2 (9.8)	43.4 (9.9)	39.2 (10.6)
Country of birth (%)				
UK	66 (80)	23 (88)	20 (71)	23 (82)
Non-UK	15 (18)	2 (8)	8 (29)	5 (18)
Didn't say	1 (1)	1 (4)	0 (0)	0 (0)
Relationship status (%)				
Single	11 (13)	7 (27)	1 (4)	3 (11)
In a relationship	8 (10)	2 (8)	2 (7)	4 (14)
Living with partner/married	54 (66)	14 (54)	21 (75)	19 (68)
Separated, divorced, widowed	7 (9)	3 (12)	2 (7)	2 (7)
Prefer not to say	2 (2)	0 (0)	2 (7)	0 (0)
Fluency of spoken English (%)	82 (100)	26 (100)	28 (100)	28 (100)
Fluency of written English (%)	82 (100)	26 (100)	28 (100)	28 (100)

*Work characteristics*				
Organisation (%)				
A	7 (9)	2 (8)	1 (4)	4 (14)
B	12 (15)	4 (15)	3 (11)	5 (18)
C	17 (21)	4 (15)	5 (18)	8 (29)
D	36 (44)	13 (50)	16 (57)	7 (25)
E	3 (4)	1 (4)	1 (4)	1 (4)
F	7 (9)	2 (8)	2 (7)	3 (11)
Occupation (%)				
Modern professional occupations	15 (18)	9 (35)	2 (7)	4 (14)
Clerical and intermediate occupations	21 (26)	7 (27)	3 (11)	11 (39)
Senior managers or administrators	39 (48)	9 (35)	18 (64)	12 (43)
Technical and craft occupations	4 (5)	1 (4)	2 (7)	1 (4)
Traditional professional occupations	3 (4)	0 (0)	3 (11)	0 (0)
Years in paid employment (SD)	19.7 (10.5)	19.0 (9.7)	20.9 (11.5)	19.0 (10.5)
Income in £ per year (%)				
10,000–19,000	19 (23)	7 (27)	4 (14)	8 (29)
20,000–29,000	25 (30)	6 (23)	9 (32)	10 (36)
30,000–39,000	22 (27)	5 (19)	12 (43)	5 (18)
40,000–49,000	12 (15)	7 (27)	3 (11)	2 (7)
50,000–59,000	1 (1)	0 (0)	0 (0)	1 (4)
Prefer not to say	3 (4)	1 (4)	0 (0)	2 (7)

*Education level*				
Highest qualification (%)				
Masters, Doctorate or equivalent	32 (39)	15 (58)	12 (43)	5 (18)
First degree or equivalent	34 (41)	8 (31)	12 (43)	14 (50)
A level or equivalent	9 (11)	2 (8)	0 (0)	7 (25)
GCSE Grade A*–C or equivalent	7 (9)	1 (4)	4 (14)	2 (7)

*Experience*				
Familiarity with the online environment (%)				
Very	43 (52)	16 (62)	14 (50)	13 (46)
Fairly	32 (39)	8 (31)	12 (43)	12 (43)
Moderate	6 (7)	2 (8)	2 (7)	2 (7)
A little experience	1 (1)	0 (0)	0 (0)	1 (4)
Recent diagnosis of mental illness %	40 (49)	11 (42)	13 (46)	16 (57)
Currently taking medication for anxiety or depression %	27 (33)	9 (35)	9 (32)	9 (32)
Previous training on stress management %	39 (48)	10 (38)	12 (43)	17 (61)

Notes

DG = Discussion Group; MSG = Minimal Support Group; WLC = Wail List Control.

The average age of participants was 41.0 (SD 10.2). The majority were female (70/82, 85%), were born in the UK (66/82, 80%), were married or living with a partner (54/82 66%), were in senior manager or administrator roles (39/82, 48%; as described by the UK National Statistics Socio-Economic Classification), and had at least a first degree (66/82, 80%). Participants had been in paid employment for a mean of 19.7 (SD 10.5) years. All were fluent in both written and spoken English. Most (75/82, 91%) were fairly or very familiar with the online environment. Just under half of participants (40/82, 49%) had a recent diagnosis of mental illness, with 33% (27/82) currently taking medication for anxiety or depression. Previous experience of stress management training was reported by 48% (39/82) of participants. Participants were asked on a scale of 1 to 10 (with 1 = *not important at all*, and 10 = *very important*) how important is was to them to reduce their level of workplace stress. Over 87% of participants (71/82) indicated 8 or above, with 51% (42/82) indicating the highest score. Two of the six organisations that participated in this study provided demographic information. Comparing gender information, a larger number of females participated in the study than were in the workforce (organisation 2: 52% female in the organisation, 83% of participants in the study female. Organisation 3: 67% female in the organisation, 88% of participants in the study female).

### 3.3 Engagement outcomes

One univariate outlier was found on each of the login and the page view variables; these were replaced with the group mean in each case. Sensitivity analysis indicates that if the outliers were not removed then the effect sizes remain in the same order of magnitude as reported below, but the CI for both the mean number of logins and the mean number of pages viewed no longer cross zero.

Data for the primary and secondary engagement measures are shown in Table 2. The mean for each of the three engagement outcomes show a greater number of logins, modules completed and page views for the DG compared to the MSG. A medium between group effect size was observed for the primary outcome of login (*d* = 0.51; 95% CI: − 0.04, 1.05) and for secondary outcome page views (*d* = 0.53; 95% CI: − 0.02, 1.07), and a small effect size (*d* = 0.26; 95% CI: − 0.28, 0.80) was observed for modules completed. Confidence intervals for all outcome effect sizes crossed zero. No difference was found in the self-report engagement between the two groups.

**Table 2:** Primary and secondary outcome: Engagement of WorkGuru.

Outcome	DG (*n* = 26)			MSG (*n* = 28)			
	M	SD	Range	M	SD	Range	Cohen's *d* (95% CI)
Logins	9.4	7.3	0–25	5.8	6.8	0–26	0.51 (− 0.04, 1.05)
Modules completed	2.2	2.9	0–10	1.5	2.4	0–9	0.26 (− 0.28, 0.80)
Page views	143.1	117.6	0–410	83.2	107.6	0–441	0.53 (− 0.02, 1.07)
Self-report engagement	3.18	1.13	1–5	3.35	1.17	1–5	0.15 (− 0.68, 0.39)

### 3.4 Psychological outcomes

Descriptive data for both psychological outcomes at all three assessment points is shown in Table 3. Table 4 shows the between group effect sizes. At T2 a small between group effect size difference was found between both active conditions compared with the WLC on all three sub-scales of the DASS. No difference was found between the two active conditions. At T3 a small effect size difference was maintained between DG and the WLC on both the anxiety and stress subscales, and a small or medium between group effect size difference was maintained between MSG and WLC on all three subscales. Confidence intervals for all outcome effect sizes on the DASS with the exception of the T3 between group effect size between the MSG and WLC on the stress subscale, cross zero.

**Table 3:** Mean and standard deviation for the psychological outcomes (ITT sample).

	T1			T2			T3		
	DG (*n* = 26)	MSG (*n* = 28)	WLC (*n* = 28)	DG (*n* = 26)	MSG (*n* = 28)	WLC (*n* = 28)	DG (*n* = 26)	MSG (*n* = 28)	WLC (*n* = 28)
	M (SD)	M (SD)	M (SD)	M (SD)	M (SD)	M (SD)	M (SD)	M (SD)	M (SD)
*DASS*^a^									
Depression	19.9 (10.2)	20.2 (9.6)	20.5 (9.4)	16.0 (10.1)	15.1 (9.9)	18.0 (11.0)	15.5 (8.5)	13.8 (9.5)	16.0 (9.9)
Anxiety	10.8 (7.4)	12.4 (8.6)	13.6 (8.4)	10.2 (7.7)	9.3 (6.3)	12.7 (8.6)	8.8 (6.4)	7.9 (6.9)	11.0 (9.6)
Stress	23.3 (7.7)	24.0 (9.4)	24.1 (8.0)	19.8 (9.2)	19.3 (6.6)	22.4 (7.6)	18.1 (7.7)	15.9 (6.6)	20.6 (8.7)

*IWP*^b^									
Enthusiasm	8.6 (2.8)	8.4 (3.5)	7.9 (2.4)	9.7 (3.5)	9.8 (3.7)	8.6 (3.7)	9.3 (3.7)	10.0 (4.0)	9.3 (4.3)
Anxiety	14.9 (5.5)	13.7 (5.2)	14.2 (6.1)	15.8 (5.7)	15.8 (5.6)	16.1 (5.7)	17.6 (5.5)	18.7 (5.7)	16.3 (5.9)
Comfort	7.4 (2.2)	7.6 (2.7)	7.7 (2.3)	8.6 (3.2)	8.6 (3.2)	7.9 (3.0)	9.5 (3.3)	11.0 (5.1)	9.0 (3.7)
Depression	18.0 (5.7)	17.0 (5.3)	17.8 (5.1)	18.7 (5.8)	19.3 (6.5)	19.3 (5.7)	19.7 (6.3)	20.7 (6.0)	20.0 (6.2)

a Lower scores = higher wellbeing.

b Higher scores = higher wellbeing.

**Table 4:** Between groups effect sizes for psychological outcomes (ITT sample).

	T2 between group effect			T3 Between group effect		
	Cohen's d (95% CI)					
	DG & WLC	MSG & WLC	DG & MSG	DG & WLC	MSG & WLC	DG & MSG
*DASS*						
Depression	0.19 (− 0.35, 0.72)	0.28 (− 0.25, 0.80)	0.09 (− 0.45, 0.62)	0.05 (− 0.48, 0.59)	0.23 (− 0.30, 0.75)	0.19 (− 0.35, 0.72)
Anxiety	0.31 (− 0.24, 0.84)	0.45 (− 0.09, 0.97)	0.13 (− 0.41, 0.66)	0.27 (− 0.27, 0.80)	0.37 (− 0.16, 0.89)	0.14 (− 0.67, 0.40)
Stress	0.31 (− 0.23, 0.84)	0.44 (− 0.10, 0.96)	0.06 (− 0.60, 0.47)	0.30 (− 0.24, 0.84)	0.61 (0.06, 1.14)	0.31 (− 0.84, 0.23)

*IWP*						
Enthusiasm	0.30 (− 0.84, 0.23)	0.32 (− 0.20, 0.85)	0.03 (− 0.51, 0.56)	0 (− 0.53, 0.53)	0.17 (− 0.36, 0.69)	0.18 (− 0.35, 0.72)
Anxiety	0.05 (− 0.59, 0.48)	0.05 (− 0.58, 0.47)	0.00 (− 0.53, 0.53)	0.23 (− 0.31, 0.76)	0.41 (− 0.12, 0.94)	0.20 (− 0.34, 0.73)
Comfort	0.23 (− 0.76, 0.31)	0.23 (− 0.75, 0.30)	0 (− 0.53, 0.53)	0.14 (− 0.68, 0.39)	0.45 (− 0.98, 0.08)	0.35 (− 0.19, 0.88)
Depression	0.10 (− 0.64, 0.43)	0.00 (− 0.52, 0.52)	0.10 (− 0.44, 0.63)	0.05 (− 0.58, 0.49)	0.00 (− 0.52, 0.52)	0.16 (− 0.37, 0.70)

At T3, small between group effect size differences were found between the two active conditions on both the depression and the stress subscales. Examination of the means suggests that the means for both depression and stress are smaller in the MSG.

Findings from the IWP data suggest that there was a small effect size difference between both active conditions and WLC on the enthusiasm and comfort subscales at T2, which is maintained in the MSG group at T3, suggesting that there is an increase in enthusiasm and comfort in the active conditions and that this is maintained at T3 in the MSG group. Contrary to the DASS data, an effect size of zero or only a very small effect size was found on the depression and the anxiety subscales at T2. At T3 a small effect size difference is found on the anxiety subscale between both active conditions and the WLC. Small group effect sizes are also found at T3 between the two active conditions on both the anxiety and the comfort subscales. Examination of the means suggests that the improvements to both anxiety and comfort are in favour of the MSG group. Confidence intervals for all outcome effects sizes on the IWP measure crossed zero.

### 3.5 Per-protocol analysis

Per-protocol analysis was conducted using data from participants who had logged into the program ≥ 3 times, and who had completed questionnaires. Protocol adherence was achieved by 70% of participants. Per-protocol analysis mirrored the effect size for the primary outcome number of logins (*d* = 0.42, 95% CI: − 0.22, 1.05). Results for the DASS showed larger effect sizes: at T2 a medium to large between group effect size was found between both active conditions and the WLC on all subscales of DASS, small to medium effect sizes were maintained at T3. The between group effect sizes for MSG and WLC at both T2 and T3 for the subscale stress were both significant effect sizes (T2: *d* = − 0.76, 95% CI: − 1.41, − 0.09; T3: *d* = − 0.64, 95% CI: − 1.25, − 0.01). The confidence intervals for all the other effect sizes crossed zero. At T3 a small to medium between group effect size was found between both the active conditions with the mean scores showing a lower level of depression, anxiety and stress for the MSG, confirming the findings in the ITT analysis that while participants in both active conditions have reduced levels of stress, depression and anxiety, participants in the MSG seem to benefit most from the intervention.

Per-protocol analysis of the IWP data were consistent with the ITT analysis but showed larger effect sizes: a medium effect size difference was found between both active conditions and the WLC on both the enthusiasm and comfort subscales, at T3 a small effect size was maintained between MSG and WLC, confirming the finding that there was an increase in enthusiasm and comfort in the active conditions and that this was maintained in the MSG group at T3. At T3 a small to medium effect size was seen on all the subscales between the MSG and WLC. Examination of the means show an increase in enthusiasm and comfort and a decrease in depression and anxiety in favour of the MSG. A small effect size difference was found on all the subscales at T3 between the two active conditions. The mean scores confirm the ITT findings that participants in the MSG seemed to benefit most from the intervention. Confidence intervals for all outcome effect sizes on the IWP measure crossed zero.

### 3.6 Client satisfaction, usability, acceptability and credibility

At T2 all of the 17 participants in the DG and only 17 of the 20 participants in the MSG group who provided data competed the client satisfaction and system usability questionnaires. Client satisfaction with WorkGuru was high, with 82% (14/17) in the MSG and 71% (12/17) in the DG rating the service that they had received as excellent or good. The majority of participants said that they had got the kind of service that they wanted (76% in both groups 13/17), and that they would recommend the program to a friend (MSG: 65% 11/17; DG: 76% 13/17). Participants in the MSG were more satisfied with the amount of help that they received (MSG: 76% 13/17; DG: 59% 10/17) and their general satisfaction with the service appeared to be higher (MSG: 76% 13/17; DG 65% 11/17). They were more likely to say that the service helped them to deal with their problems (MSG: 76% 13/17; DG 53% 9/17) and that they would come back to WorkGuru if they needed help again (MSG 71% 12/17; DG 47% 8/17). A small number of participants (MSG: 12%, 2/17; DG 18%, 3/17) said that none of their needs had been met, and one participant (6%) in the DG said that the service seemed to have made their problems worse. The mean system usability score for DG was 68.4 (SD 15.8) and for MSG 76.0 (SD 13.5).

Participants in both active conditions were given the CEQ at 2 weeks from randomisation. Intervention credibility and expectancy of participants about improvements was similar across both groups (mean credibility for DG = 15.4 (SD = 3.7) and for the MSG = 16.3 (SD 3.9); mean expectancy for DG = 12.2, (SD = 5.2) and for the MSG = 14.8 (SD = 5.5)).

### 3.7 Sickness absence

**Fig. 2:**
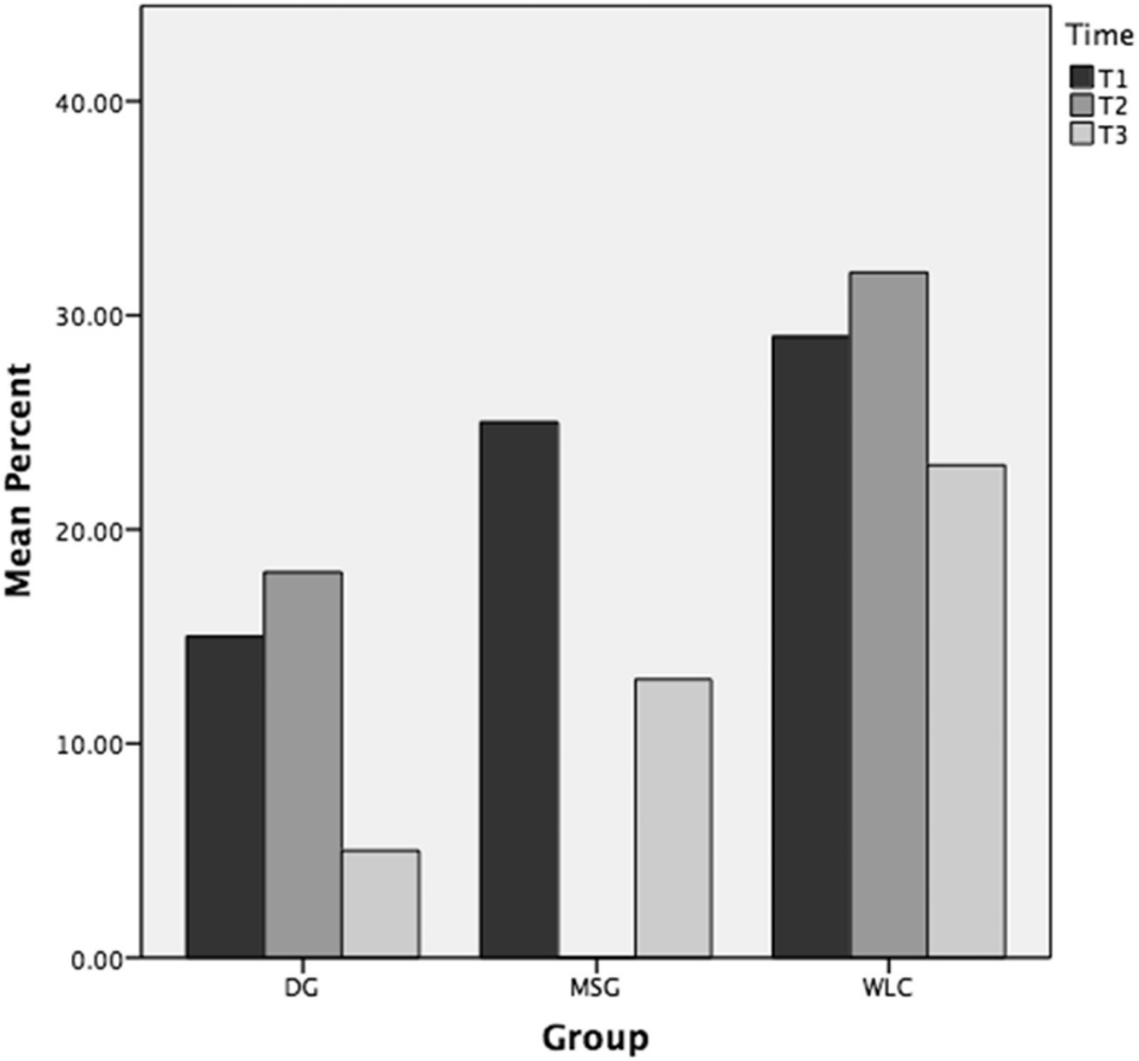
Have taken time off sick for stress related complaint in last 8 weeks.

Participants were asked at all three time points if they had taken time off sick for a stress related complaint in the last eight weeks. All groups had seen a fall between T1 and T3 in the number of participants who had been absent from work. For the DG the mean at T1 was 15% (4/26), at T2 18% (3/17), and at T3 5% (1/22). For the MSG it was T1 25% (7/28), at T2 0% (0/28), and T3 13% (3/23). For the WLC it was T1 29% (8/28), at T2 32% (8/25) and T3 23% (6/26). Fig. 2 shows the self-report sickness absence for stress related complaints.

### 3.8 Care as usual

Self-reported care as usual was examined to see if there were any differences between the three groups at the three time points. Participants accessed a range of support for their mental health problems including from GPs, counsellors, online self-help (e.g. a website for information), psychiatrists, psychologists, occupational health nurses and doctors. No differences were found between the groups on the number or type of support accessed, or the number of participants who had been prescribed medication for anxiety or depression. A similar number of participants across the groups reported accessing online support for information.

### 3.9 Moderator analysis

Possible moderators of engagement were explored. The means for participants on goal conflict, time pressure, job autonomy and level of psychological distress (total of DASS subscales) at baseline were calculated and the participants placed in groups depending on whether they were above or below that mean. Table 5 shows the mean number of logins for each of the groups and the between group effect sizes. The analysis showed a small effect size for goal conflict (*d* = 0.22, 95% CI: − 0.75, 0.32), time pressure (d = 0.19, 95% CI: − 0.73, 0.35) and level of psychological distress (*d* = 0.43, 95% CI: − 0.93, 0.12) at baseline. Examination of the means suggested that participants who reported lower goal conflicts, lower time pressure and lower psychological distress at baseline had a higher number of logins to the stress management program. No effect size difference was found between the two groups for job autonomy. Confidence intervals for all moderator analysis effect sizes crossed zero.

**Table 5:** Moderator analyses.

Moderator (*n*)	Mean number of logins	SD	Cohen's *d* (95% CI)
*Goal conflict*			
Conflicted (26)	6.7	5.8	0.22 (− 0.75, 0.32)
Non-conflicted (28)	8.3	8.4	

*Time pressure*			
Time pressured (22)	6.7	6.0	0.19 (− 0.73, 0.35)
Not time pressured (32)	8.1	8.0	

*Job autonomy*			
Autonomous (30)	7.5	5.7	0.00 (− 0.54, 0.54)
Non autonomous (24)	7.5	8.9	

*Level of psychological distress at baseline*			
Higher distress (33)	6.3	6.2	0.43 (− 0.98, 0.12)
Lower distress (21)	9.4	8.4	

### 3.10 Exploratory analyses

Further exploratory inferential analysis was conducted on per-protocol data. No significant differences were found in *t*-tests between the active conditions on the number of logins, page views, messages sent by and to the coach and the number of modules completed. The ANOVA showed a significant effect of intervention on levels of stress at T2: *F*(2, 53) = 3.19, *p* = 0.049. Contrasts show that stress levels were significantly different for participants in both DG (*t* (53) = 2.0, *p* = 0.050) and MSG (*t* (53) = 2.2, *p* = 0.033) compared to WLC. This difference was maintained at T3 in MSG (*t* (59) = 2.2, *p* = 0.032). No other significant difference was found on the psychological measures.

### 3.11 Discussion group

Two eight-week guided discussion groups were delivered via a bulletin board. The first group had 16 participants and the second group had 10. The second group started five weeks after the first group started. The bulletin board was viewed 493 times by participants (M = 19.0, SD = 19.9) and 99 contributions were made: 57 by participants and 42 by the coach. The mean number of contributions made per participant was 2.2 (SD = 2.4). An approximation of the time spent by the coach on each contribution that she made is 15 min; additionally approximately 30 min per week was spent by the coach logging in and monitoring each of the groups. This equates to just over 5 h per group spent by the coach in contributing to the discussion and 4 h per group on monitoring, which is slightly > 1 h of coach time per group per week or 41.5 min per participant across the eight-weeks.

Results from the online support group questionnaire (Table 6) in which items were rated on a score of 1–10 where 1 means *not at all* and 10 means *very much*, indicated that participants were not very satisfied with the groups. Only two items rated at over 5 these were agreement that participants preferred to use aliases, and the relevancy of the topics chosen by the coach.

**Table 6:** Means and standard deviations of the DG's online support group questionnaire.

Subscale	M	SD
Support		
Felt supported by other members	3.1	2.3
Felt listened to by other members	3.2	2.2
Relevance		
Contributions of other group members were relevant	4.1	2.9
Topics of coach is relevant	5.1	2.8
Others addressed issues I raised	2.9	1.9
Comfort-connection		
Comfortable contributing to group	4.7	3.3
Felt connection to other members	2.4	1.7
Satisfied with being part of a group	3.1	2.2
Prefer aliases to real identities	5.6	3.8
Total	3.8	2.3

Note: Items scored on a range from 1 (*not at all*) to 10 (*very much*). *n* = 14.

### 3.12 Coach activity

During the course of this study, across both active conditions combined, the coach sent 185 individual coaching messages through the secure system (M = 3.6, SD = 1.1) and received 43 messages (M = 0.8, SD = 1.6) from participants. The content of the messages sent from participants were: acknowledging contact from the coach (*n* = 16), reflecting on the content of the modules (*n* = 12), sharing assignments (*n* = 5) asking a technical question (*n* = 4), requesting extended access to the site (*n* = 2), explaining absence (*n* = 2), and questions about the research (*n* = 2). Messages sent by the coach at initial log-on, two weeks and six weeks were based on a template, but personalised where possible. All responses to enquiries initiated by participants were personalised. An approximation of time spent by the coach on each message is 5 min, this equates to 15.4 h across the 8-week course spent by the coach on sending messages to participants in both the active conditions. The coach spent 18.7 min per participant sending, reading and responding to messages from the DG, and 17.0 min per participant in the MSG group.

In the DG (*n* = 25) the mean number of messages sent by the coach directly to participants (not through the bulletin board) was 3.7 (SD = 1.1), and in the MSG (*n* = 27) it was 3.4 (SD = 1.1). In the DG the mean number of messages sent by participants to the coach was 1.3 (SD = 1.9), in the MSG it was 0.37 (SD = 1.0). There is a small between group effect size for the number of messages sent by the coach (*d* = 0.28, 95% CI: -0.27, 0.82) and a medium between group effect size for the number of messages sent by participants (*d* = 0.62, 95% CI: 0.07, 1.18) suggesting that more messages are sent by both the coach and participants in the discussion group.

### 3.13 Negative effects

Participants were asked what if any positive or negatives effects were caused by being in an active condition or being in the control. Across both T2 (*n* = 17) and T3 (*n* = 21) participants in the DG identified eight positive effects and 13 negative effects (this included duplication where participants made the same comment at both time points). Across both T2 (*n* = 20) and T3 (*n* = 23) participants in the MSG identified 9 positive effects and 7 negative effects (this included duplication). Across both T2 (*n* = 25) and T3 (*n* = 26) the WLC identified 3 negative effects (this included duplication). Positive effects included: *It made me think*/*know myself better* (*n* = 7), and: *I liked the support from the coach/community* (*n* = 3). Negative effects included: *I didn't have time to complete it* (*n* = 8), *I found it stressful* (*n* = 5) and: *I felt guilty for not using it enough* (*n* = 3). The negative effects of being in the control were: *Disappointment at being in the control* (*n* = 2) and: *Not having any contact with the coach* (*n* = 1).

### 3.14 Contamination

The extent of contamination between the groups was monitored by asking the extent to which participants had discussed the research with colleagues in other groups. At T2 94% (58/62) of participants said *not at all* and 6% (4/62) said *a little bit*. At T3 87% (62/71) said *not at all* and 13% (9/71) said *a little bit.*

## 4 Discussion

### 4.1 Principal findings

Results of this study support the effectiveness of an online facilitated discussion group in increasing the number of logins to a minimally supported digital stress management program. Medium between group effect sizes were found for both logins and page views, and a small effect size for the number of modules completed. No difference was found in self-reported engagement between the groups. Both the numbers of logins and page views seem to be a more sensitive measure of physical engagement with the program, but metrics such as login and page views may not necessarily measure the extent to which participants are psychologically engaged; clicking through a large number of pages may be a sign of disengagement as participants are not necessarily taking the time to engage psychologically with the content of the page. Self-report measures may be a more useful measure of engagement as they provide the user's assessment of their experience (O'Brien and Toms, 2009), but it is unlikely that the one-item self-report engagement measure developed for this study is sensitive enough to give a meaningful measure of the individual's experience.

### 4.2 Psychological outcomes

Results from this study suggest that the trend appears to be that access to the web-based stress management intervention is associated with lower levels of depression, anxiety and stress, and an increase in comfort and enthusiasm compared with the control condition and that these outcomes are largely maintained at follow-up. Participants who accessed the intervention without the discussion group seem to have potentially derived greater benefit. Per-protocol analysis confirms these findings. Further research may usefully explore this possibility by examining the influence of engagement within the individual groups. The effect sizes for the DASS outcomes in this study are in line with those reported in recent meta-analyses on digital stress management interventions (Heber et al., 2017) and digital mental health interventions delivered in the workplace (Carolan et al., 2017).

### 4.3 Satisfaction, usability, acceptability and credibility

Satisfaction with the intervention, and intervention usability was higher in the MSG than the DG. The intervention credibility and the expectancy of participants about improvements were similar across both active conditions, but satisfaction with the discussion groups was low. When recruiting to the study the intention was to run one discussion group of 30 participants (Carolan et al., 2016). The size of the discussion group was based on previous experience at WorkGuru that suggested that a group of 30 optimised participant engagement. Because of the time that it was taking to recruit to the study, the decision was made to run two groups so that participants would not have to wait for more than three weeks for their group to start. When the group had started, new recruits were still able to join the group over the first two weeks. The smaller size of the groups, the delay in the groups starting, and the experience of participants joining the groups after they had started may have impacted on both the satisfaction with the groups, and the effectiveness of the groups in optimising engagement. Because of these problems with the study design we would suggest that our findings that participants accessing the intervention without a discussion group benefited most from the intervention be interpreted with caution, and that further research is conducted to examine the optimum size and other optimising factors for online facilitated discussion groups delivered alongside digital minimum support interventions.

### 4.4 Moderator analysis

A small effect size difference was found between participants that reported both higher and lower levels of goal conflict, higher and lower levels of time pressure, and higher and lower levels of psychological distress at baseline. Examination of the means suggested that participants who reported lower goal conflicts, lower time pressures and lower distress login to the intervention more frequently. Organisations participating in this research were encouraged to offer participants 1 h a week to complete the program. Employers were not aware of which of their employees were participating in the study so it is unlikely that this message was reinforced to individual participants. Future research could look at whether within an occupational setting, prioritising and setting aside time for individual employees to access digital mental health programs increases the number of times that participants login to the intervention.

### 4.5 Explorative analysis

The explorative inferential analysis confirmed our finding that access to the intervention resulted in a significant reduction in levels of stress at T2 and that this was maintained in the MSG at T3. In recognition that this is a pilot study, we suggest caution in interpreting these findings.

### 4.6 Coach activity

For both the active conditions combined the coach spent a total of 15.4 h sending messages and responding to messages from participants, an additional nine hours per group was spent by the coach monitoring and contributing to the on-line discussion groups. If you combine the amount of coach time spent per participant in facilitating the two discussion groups (41.5 min) with the time spent per participant sending, reading and responding to messages (DG = 18.7; MSG = 17.0) then each DG participant required a mean of 60.2 coaching minutes, and each MSG participant required a mean of 17.0 min. Group means and between group effect sizes show that more messages (outside of the bulletin board) were sent between the coach and participants in the DG compared to the MSG suggesting that the additional time spent by the coach facilitating the discussion group does not result in less individual messages being sent; the discussion group may generate additional individual contact with the coach.

### 4.7 Negative effects

Participants were asked what if any negative effects were caused by being in the group that they were allocated to. Participants in the DG identified almost twice as many negative effects of being in the group than the MSG. Some participants felt that the demands of the web-based program increased their feelings of stress as they felt guilty for not using the program enough, or felt that they didn't have time to complete it. Being in the group that accessed WorkGuru alongside a discussion group seems to have added to that strain. Further research is needed to gain a greater understanding of the extent to which the workplace is a suitable environment for delivering digital mental health programs. Do the benefits of digital mental health that have been identified in community and health settings (e.g. the ability to access at a time and at a pace that is convenient to the user) translate as benefits in an occupational setting? Or are there additional challenges to delivering these interventions in the workplace (e.g. stigma, time pressure, competing priorities) that need to be overcome?

### 4.8 Learning from this pilot

This pilot study has enabled us to make a more confident but still tentative prediction of effect size for our primary outcome of engagement, we recognise however the limitations of using this effect size to determine sample size for a full trial (Leon et al., 2011). The pilot supports optimal adherence to the intervention as being ≥ 3 logins, and it supports the number of login and page views as being a useful measure of exposure to the intervention. Module completion does not appear to be a useful measure; this may be because exposure to anything < 100% of the module would not register as module completion whereas participants may benefit from the module without having visited every page. A subjective measure of engagement does appear to be useful, but a more comprehensive measure than the one item measure for this pilot should be used. IWP does not seem to be a measure that is sensitive to the between group changes intended by this CBT based stress management program, a future study should explore using an alternative measure of occupational outcome (e.g. work engagement or productivity).

One of the challenges of running this pilot study was the recruitment of organisations; out of 780 invitations to individuals to nominate their organisation to participate in the study, 19 organisations expressed an interest and six organisations were recruited. One explanation for this low take-up by organisations may be that the individuals on the mailing list were not in the position of authority or influence needed to put forward their organisation for the research. Between them, the six organisations taking part in the study recruited 84 participants; a future study may need to spend more time with organisations supporting them to maximise their recruitment of participants. Thought also needs to be given to recruiting into the discussion groups in order to minimise the wait for the groups to start and to ensure that a larger number of participants are recruited to each group. Increasing the speed of recruitment may provide a solution.

### 4.9 Limitations

This study had a number of limitations. The first was a limitation of randomising at the level of the individual, which is the potential for contamination between groups: participants in the active conditions discussing the content of the intervention with the WLC. There is no evidence of contamination at T2 but there is some evidence that between group conversations had taken place at T3. A second limitation was the generalisability of our findings: participants recruited to this study were volunteers who had increased levels of stress, and were predominantly well educated females working in social care or the knowledge industry in senior manager or administrator roles, this is not representative of the general workforce. There is a strong need for future research on occupational digital mental health interventions to target industries and occupations that are traditionally under represented in these studies, this includes employees working in blue-collar roles. Only two of the three participating organisations were able to provide demographic data to make a comparison between their workforce, and employees recruited to the study. This information was further limited by a difference between the metrics used by organisations and the metrics used in this study. Future research should work with organisations to collect comparable demographic data so that a better comparison can be made between the workforce and study participants. A third limitation was the recruitment of a targeted population: participants with elevated levels of stress. Targeting these interventions towards individuals who are perceived to be experiencing stress may add to the stigma of mental health programs impacting on reach and up-take. Future studies may wish to evaluate similar programs with universal populations. Fourthly, some of the measures used in this study were developed or adapted for the study (i.e. the acceptability and the goal conflict measures), and were found to have relatively low reliability, which may impact on the strength of our findings. Fifthly, a failure in randomisation in the occupational groups could have affected the outcomes; we would expect a larger study to correct that. Sixthly, the measures of engagement used in this study were (with the exception of a limited self-report measure) confined to measures of exposure (i.e. number of login and pages viewed) future studies of occupational digital mental health interventions may wish to utilise more comprehensive measures of program engagement. Finally, we recognise the limitations of generalising conclusions from this pilot study and would suggest caution in interpreting our findings.

### 4.10 Conclusions

The findings of this study suggest that access to an online facilitated discussion group increases engagement with a minimally support occupational digital mental health intervention (as defined by number of logins) but that this increase does not necessarily result in improved psychological outcomes or increased satisfaction when compared to access to the CBT based stress management intervention on its own. Access to the stress management program resulted in lower levels of depression, anxiety and stress and an increase in comfort and enthusiasm post intervention that were largely maintained at follow-up.

## Conflict of interest statement

SC is the founder of WorkGuru, and continues to have a commercial interest in the company.

## Authors' contributions

SC is the principal investigator for the study. SC and KC conceptualised the initial trial design. This was developed with the help of PRH and KG. SC drafted the manuscript and conducted the data analysis. KC, PRH and KG provided feedback and contributed to the final version of the manuscript. All authors have read and approved the manuscript.
